# Patient- and Clinic Visit-Related Factors Associated with Potentially Inappropriate Medication Use among Older Home Healthcare Service Recipients

**DOI:** 10.1371/journal.pone.0094350

**Published:** 2014-04-10

**Authors:** Chirn-Bin Chang, Hsiu-Yun Lai, Shu-Yu Yang, Ru-Shu Wu, Hsing-Cheng Liu, Hsiu-Ying Hsu, Shinn-Jang Hwang, Ding-Cheng Chan

**Affiliations:** 1 Department of Internal Medicine, Changhua Christian Hospital, Changhua, Taiwan, School of medicine, Kaohsiung Medical University, Kaohsiung, Taiwan, School of medicine, Chung Shan Medical University, Taichung, Taiwan; 2 Department of Family Medicine, National Taiwan University Hospital, Hsinchu, Taiwan; 3 Department of Pharmacy, Taipei City Hospital, Taipei, Taiwan; 4 Taipei City Psychiatry Center, Taipei City Hospital, Taipei, Taiwan; 5 Department of Family Medicine, Taipei Veteran General Hospital, Taipei, Taiwan; 6 Department of Geriatrics and Gerontology, and Department of Internal Medicine, National Taiwan University Hospital, Taipei, Taiwan; University of Glasgow, United Kingdom

## Abstract

**Objectives:**

Taiwanese National Health Insurance (TNHI) provides home healthcare services to patients with skilled nursing needs who were homebound or lived in nursing homes. Studies on potentially inappropriate medications (PIMs) for older home healthcare service recipients (HHSRs) are growing, but comparisons among newer criteria of PIMs have not been applied. The aim of this study was to explore the prevalence and correlates of PIMs based on three different instruments published after 2010 among older HHSRs.

**Materials and Methods:**

We performed cross-sectional analysis of the TNHI Research Database. A total of 25,187 HHSRs aged more than 65 years in 2009 were included. Medication lists independent of chronic conditions from the 2012 Beers criteria, PIM-Taiwan criteria, and the PRISCUS (Latin for “old and venerable”) criteria were used. Analysis was performed separately at patient and clinic-visit level. T-tests, chi-square analysis, and multivariate logistic regressions were used where appropriate.

**Results:**

The prevalence of having at least one PIM at patient and clinic-visit level was highest with the Beers (82.67%, 36.14% respectively), followed by the PRISCUS (68.49%, 25.13%) and PIM-Taiwan (63.04%, 19.21%) criteria. At patient level, polypharmacy (odds ratio (OR) 2.53 to 4.90), higher number of clinic (OR 1.15 to 1.41), hospital (OR 1.24 to 1.64), and physician (OR 1.15 to 1.41) visits were associated with PIM use for all 3 sets of criteria. At clinic-visit level, internist/family physicians (OR 1.26 to 1.72) and neurologists/psychiatrists (OR 1.73 to 5.87) were more likely to prescribe PIMs than others. Psychotropic drugs and first generation antihistamines accounted for most of the top ten PIMs.

**Conclusion:**

The prevalence of PIMs was high among older Taiwanese HHSRs. Polypharmacy and certain medical specialties were associated with a higher likelihood of PIM prescriptions. Provider education and medication review and reconciliation should be considered.

## Introduction

The demands of long-term care services including home healthcare have been rising in many countries, including Taiwan [1]. The National Health Insurance (NHI) system reimburses home healthcare services (HHS) in Taiwan. A patient can apply for HHS when he or she has the following conditions: limited self-care ability (need assistance more than 50% of the time when the patient is awake or chair-bound/bed-bound), with needs of specialized care (nasogastric tube, Foley catheter, tracheostomy tube or chronic wound), nursing care for chronic diseases, or continuity of nursing care after acute illness [1]. These services are delivered by facilities and providers with special qualifications. In Taiwan, the services were not limited to community patients. Nursing home residents may also receive HHS if they meet the enrolment criteria. Home healthcare service recipients (HHSRs) usually have complex chronic diseases and a higher level of dependence for activities of daily living [2].

Pharmacological therapy is often used to treat chronic diseases in older adults, and HHSRs often visit multiple providers for prescriptions for their multiple chronic conditions. In addition, the pharmacokinetics and pharmacodynamics of these drugs change with age or the progression of chronic diseases. These changes and polypharmacy lead to a higher incidence of adverse drug events, which have been reported to be an important cause of hospitalization, morbidity, and mortality for older patients [3]–[5]. Some of these events are considered preventable by avoiding prescribing potentially inappropriate medications (PIMs) [6]. PIM lists were developed to avoid prescribing high-risk medications for older adults. Many countries developed their own PIM lists because of country-specific approved drugs, and specific prescribing and therapeutic guidelines [7]. The PIM-Taiwan criteria were established in 2012 and were based on seven established criteria [8]. In the same year, the Beers criteria were revised [9]. In Europe, the PRISCUS (Latin for “old and venerable”) criteria were published in 2010 [10]. Although many sets of PIM criteria are available, relatively few studies have used these newer lists to detect PIMs in older HHSRs. Most of these studies have explored prevalence of PIMs and their correlates among older nursing home residents [11]–[16]. The aim of this study was to compare the prevalence and correlates of the PIMs listed in the above three criteria for older HHSRs covered by the Taiwanese NHI program.

## Methods

This study was approved by the Research Ethics Committee of National Taiwan University Hospital in 2013. The patient records/information in this database was anonymized and de-identified prior to analysis. It is a secondary data analysis of medical claims from the NHI Research Database (NHIRD) in Taiwan with the observation period in 2009.

Patients were selected if they were aged 65 years or older, and if they had received at least one HHS in 2009. Data including age, gender, diagnoses, number of clinic visits, number of physician visits, and all oral medications prescribed in 2009 were collected for all enrolled patients. The type of healthcare organization, age, gender and specialty of the treating physician, and duration and costs (New Taiwan dollar) of the oral medication were collected at each clinic visit. In addition, up to three International Classification of Diseases 9th edition Clinical Modification (ICD-9 CM) codes were recorded at the time on each clinical encounter. Detailed information on the prescribed drugs including number of daily tablets, dose of each tablet and generic names were obtained to calculate the daily dose. The duration of each drug that was prescribed on the specific clinic-visit was also collected in the claim data. Information on over-the-counter drugs and other clinical data such as weight, height, and blood pressure were not available due to the nature of the NHIRD.

Because the NHIRD did not contain an exhaustive list of diagnoses for their beneficiaries, we only included the PIMs independent of chronic conditions. Identification of PIMs was based on the table 1 of PIM-Taiwan criteria [8], table 2 of the 2012 version of Beers criteria [9], and the entire PRISCUS criteria [10]. Because some medications were considered as being PIM only when the daily dose was greater than a certain amount, daily dose was calculated as total amount (tablets or caps) of one medication divided by duration of prescription. In addition, defined daily dose (DDD) of drugs having Anatomical Therapeutic Chemical (ATC) codes was used as reference to confirm the daily dose of each prescribed medication, for example, olanzapine >1 DDD in PRISCUS criteria.

**Table 1:** Basic characteristics of the home healthcare recipients.

	N(%) or average(SD)
All	25187
Gender	
Men	11333 (45.00)
Women	13854 (55.00)
Age (years)	81.40 (7.44)
65–74	5198 (20.64)
75–84	11767 (46.72)
> = 85	8222 (32.64)
Number of diagnoses/person in one year	10.81 (6.15)
<10	12236 (48.58)
> = 10	12951 (51.42)
Number of medications/person in one year	22.02 (11.96)
<20	12904 (51.23)
> = 20	12283 (48.77)
Number of clinic visits/person in one year	24.03 (16.56)
<25	15010 (59.59)
> = 25	10177 (40.41)
Number of hospital visits/person in one year	2.76 (1.76)
<3	13546 (53.78)
> = 3	11641 (46.22)
Number of physician visits/person in one year	5.69 (3.67)
<6	14169 (56.26)
> = 6	11018 (43.74)

**Table 2:** Characteristics of the three sets of explicit criteria and their performance in detecting potentially inappropriate medications.

	Beers criteria	PIM-Taiwan criteria	PRISCUS criteria
Establish year	2012	2012	2010
Country	United States	Taiwan	German
Statements*	34	24	15
Average number of PIMs/person	8.68 (8.92)	4.62 (6.64)	6.04 (7.16)
Average number of PIMs/prescription	0.46 (0.74)	0.19 (0.45)	0.28 (0.56)
No PIM/person	4365 (17.33%)	9309 (36.96%)	7937 (31.51%)
1–5 PIMs/person	7277 (28.89%)	8397 (33.34%)	6905 (27.41%)
6–10 PIMs/person	4472 (17.76%)	3303 (13.11%)	4039 (16.04%)
>10 PIMs/person	9073 (36.02%)	4178 (16.59%)	6306 (25.04%)

*Statements are those for potentially inappropriate medications without considering drug-disease or drug-syndrome interactions.

We approached our analysis from two complementary perspectives: patient and clinic-visit levels. Individuals prescribed with at least one PIM based on the three sets of PIM criteria over the study period were considered to be PIM users. Individual demographic characteristics and healthcare utilizations were investigated for associations with PIMs. At clinic-visit level analysis, all medications prescribed by a single physician for that visit were analyzed. A clinic-visit level PIM user was defined as those prescribed with at least one PIM based on three sets of PIM criteria for that visit. The characteristics of prescribing physicians and hospitals were investigated for associations with having PIM prescriptions. Bivariate analysis was performed using the *t*-test for continuous variables and χ^2^ test for categorical variables to test the correlations between PIM use and patient level or clinic-visit level characteristics. We used stepwise multivariate logistic regression models to identify the correlates of having at least one PIM at patient level and clinic-visit level after adjusting for potential confounders. All of the tests in this study were two-tailed, and significance α was set at *P*<0.05. The top ten PIMs of each set of PIM criteria were ranked by calculation of the percentage of the number of PIMs divided by total number of medications prescribed for the HHSRs in 2009. Data were analyzed using SAS for Windows version 9.2 (SAS Institute Inc., Cary, NC, USA).

## Results

In total, 25,187 HHSRs were enrolled in this study with a mean age of 81.40 years. Nearly half of the HHSRs were aged 75–84 years, and 45% were men. The mean number of diagnoses and medications prescribed in 2009 were 10.81 and 22.02, respectively. The healthcare resource utilization was high, with the mean number of clinic, physician and hospital visits being 24.03, 5.69 and 2.76, respectively (Table 1).

The three sets of PIM criteria represented the expert opinions from three different regions. In our patient and clinic-visit level analysis, the average number of PIMs detected by the Beers criteria was highest with 8.68 for each person and 0.46 for each prescription. More than one third of the patients were prescribed with more than 10 PIMs in one year based on the Beers criteria. In contrast, nearly one third of these patients had only 1–5 PIMs based on the PIM-Taiwan and PRISCUS criteria (Table 2).

In patient-level analysis, the prevalence of having at least one PIM was also highest (82.67%) with the Beers criteria. In bivariate analysis, having higher number of diagnoses, medications and having visited higher number of different physicians and hospitals were associated with having at least one PIM in all three sets of criteria (Table 3). In multivariate analysis, male HHSRs were more likely to be prescribed with PIMs in all three sets of criteria. However, older HHSRs were more likely to have PIMs in the Beers criteria only. Those who were prescribed with more than 20 different medications in 2009 were more likely to be prescribed with PIMs in all three sets of criteria (Table 4). 

**Table 3:** Patient-level bivariate analysis (N = 25187) by the presence of least one PIM according to the three sets of potentially inappropriate medication criteria.

	Beers		PIM-Taiwan		PRISCUS	
	With N (%) or Mean (SD)	Without N (%) or Mean (SD)	With N (%) or Mean (SD)	Without N (%) or Mean (SD)	With N (%) or Mean (SD)	Without N (%) or Mean (SD)
Patient number	20822 (82.67)	4365 (17.33)	15878 (63.04)	9309 (36.96)	17250 (68.49)	7937 (31.51)
Age (y)	81.4(7.38)	81.36(7.67)	81.19 (7.34)	81.76 (7.59)***	81.23 (7.32)	81.78 (7.66)***
65–74	4253(81.82)	945(18.18)	3373(64.89)	1825(35.11)***	3612(69.49)	1586(30.51)***
75–84	9788(83.18)	1979(16.82)	7516(63.87)	4251(36.13)	8197(69.66)	3570(30.34)
> = 85	6781(82.47)	1441(17.53)	4989(60.68)	3233(39.32)	5441(66.18)	2781(33.82)
Men	9487 (83.71)	1846 (16.29)***	7398 (65.28)	3935 (34.72)***	8072 (71.23)	3261 (28.77)***
Women	11335 (81.82)	2519 (18.18)	8480 (61.21)	5374 (38.79)	9178 (66.25)	4676 (33.75)
Number of diagnoses	11.69 (6.18)	6.62 (3.85)***	12.59 (6.30)	7.78 (4.46)***	11.97 (6.32)	8.30 (4.88)***
<10	8745 (71.47)	3491 (28.53)***	5648 (46.16)	6588 (53.84)***	6982 (57.10)	5254 (42.90)***
> = 10	12077 (93.25)	874 (6.75)	10230 (78.99)	2721 (21.01)	10268 (79.30)	2683 (20.70)
Medication number	24.07 (11.79)	12.25 (6.84)***	26.07 (12.00)	15.11 (8.09)***	24.79 (12.16)	16.00 (8.90)***
<20	8314 (68.93)	3747 (31.07)***	5171 (42.87)	6890 (57.13)***	6526 (54.11)	5535 (45.89)***
> = 20	12508 (95.29)	618 (4.71)	10707 (81.57)	2419 (18.43)	10724 (81.70)	2402 (18.30)
Number of clinic visits	26.03 (16.79)	14.46 (11.24)***	28.11 (17.20)	17.06 (12.65)***	26.70 (17.06)	18.22 (13.71)***
<25	11345 (75.58)	3665 (24.42)***	7765 (51.73)	7245 (48.27)***	9153 (60.98)	5857 (39.02)***
> = 25	9477 (93.12)	700 (6.88)	8113 (79.72)	2064 (20.28)	8097 (79.56)	2080 (20.44)
Number of hospital visits	2.95 (1.81)	1.85 (1.09)***	3.15 (1.88)	2.08 (1.26)***	3.01 (1.86)	2.22 (1.36)***
<3	10131 (74.79)	3415 (25.21)***	6924 (51.11)	6622 (48.89)***	8208 (60.59)	5338 (39.41)***
> = 3	10691 (91.84)	950 (8.16)	8954 (76.92)	2687 (23.08)	9042 (77.67)	2599 (22.33)
Number of physician visits	6.13 (3.74)	3.57 (2.39)***	6.59 (3.86)	4.15 (2.7) ***	6.35 (3.84)	4.25 (2.77)***
<6	10595 (74.78)	3574 (25.22)***	7210 (50.89)	6959 (49.11)***	8366 (59.04)	5803 (40.96)***
> = 6	10227 (92.82)	791 (7.18)	8668 (78.67)	2350 (21.33)	8884 (80.63)	2134 (19.37)

***P<0.0001.

**Table 4:** Multivariate logistic regression for having at least one drug as potentially inappropriate.

	Beers odds ratio	PIM-Taiwan odds ratio	PRISCUS odds ratio
Age			
65–74	1	1	1
75–84	1.07 (0.98–1.18)	0.92 (0.85–0.99)	0.98 (0.91–1.06)
> = 85	1.14 (1.03–1.25)*	0.88 (0.81–0.95)**	0.91 (0.84–0.98)**
Gender			
Male	1	1	1
Female	0.91 (0.84–0.97)**	0.86 (0.81–0.91)***	0.82 (0.77–0.87)***
Diagnosis numbers			
<10	1	1	1
> = 10	1.45 (1.30–1.61)***	1.40 (1.29–1.51)***	1.11 (1.03–1.21)*
Medication numbers			
<20	1	1	1
> = 20	4.90 (4.37–5.49)***	3.18 (2.95–3.44)***	2.53 (2.33–2.73)***
Clinic frequency			
<25	1	1	1
> = 25	1.31 (1.18–1.46)***	1.41 (1.31–1.51)***	1.15 (1.06–1.23)***
Hospital numbers			
<3	1	1	1
> = 3	1.64 (1.50–1.79)***	1.55 (1.45–1.65)***	1.24 (1.16–1.32)***
Physician numbers			
<6	1	1	1
> = 6	1.15 (1.03–1.28)*	1.16 (1.07–1.25)***	1.41 (1.31–1.52)***

*P<0.05,

**P<0.01,

***P<0.001.

In clinic-visit level bivariate analysis, having higher number and cost of medications, as well as a longer duration of prescriptions were associated with having at least one PIM in all three sets of criteria (Table 5). The proportion of PIMs detected by the PIM-Taiwan criteria per visit was lower in academic medical centers than in other healthcare organizations. Younger and male physicians were more likely to prescribe PIMs except in the PIM-Taiwan criteria. Compared with other specialties, internists/family physicians and neurologists/psychiatrists were more likely to prescribe PIMs in all 3 sets of criteria (Table 5). In multivariate logistic regression analysis, prescriptions from neurologists/psychiatrists were associated with the highest odds of having PIMs. Clinic visits in academic medical centers were only associated with a higher likelihood of PIM prescriptions in the PIM-Taiwan criteria (Table 6).

**Table 5:** Clinic visit-level bivariate analysis (N = 605239) according to the three sets of potentially inappropriate medication criteria.

	Beers		PIM-Taiwan		PRISCUS	
	With N (%) or Mean (SD)	Without N (%) or Mean (SD)	With N (%) or Mean (SD)	Without N (%) or Mean (SD)	With N (%) or Mean (SD)	Without N (%) or Mean (SD)
Overall	218749 (36.14%)	386490 (63.86%)	116269 (19.21%)	488970 (80.79%)	152091 (25.13%)	453148 (74.87%)
Medication #/clinic visit	5.32 (2.70)	3.2 (2.23)***	5.4 (2.68)	3.62 (2.48)***	5.65 (2.78)	3.39 (2.31)***
Medication duration	19.06 (10.48)	14.55 (11.01)***	16.84 (10.95)	16.03 (11.05)***	21.92 (9.04)	14.26 (10.97)***
Costs of medication (NTD)	1046.80(1850.70)	597.70 (1618.00)***	905.90 (1765.20)	725.30 (1706.40)***	1243.90 (1946.60)	597.60 (1603.60)***
Type of hospital						
Medical center	36739 (40.64)	53672 (59.36)***	16000 (17.7)	74411 (82.3)***	31410 (34.74)	59001 (65.26)***
Others	182010 (35.35)	332818 (64.65)	100269 (19.48)	414559 (80.52)	120681 (23.44)	394147 (76.56)
MD age (y)	46.87 (10.00)	47.04 (10.10)***	47.35 (10.15)	46.89 (10.04)***	45.83 (9.24)	47.37 (10.30)***
MD age < = 45	101366 (36.43)	176881 (63.57)***	50708 (18.22)	227539 (81.78)***	75920 (27.29)	202327 (72.71)***
MD age >45	117383 (35.90)	209609 (64.10)	65561 (20.05)	261431 (79.95)	76171 (23.29)	250821 (76.71)
MD gender						
Female	20668 (35.02)	38348 (64.98)***	10601 (17.96)	48415 (82.04)***	15505 (26.27)	43511 (73.73)***
Male	198006 (36.27)	347976 (63.73)	105614 (19.34)	440368 (80.66)	136522 (25.00)	409460 (75.00)
MD specialty						
Internist/family physician	116043 (36.11)	205323 (63.89)***	62231 (19.36)	259135 (80.64)***	75550 (23.51)	245816 (76.49)***
Neuro-Psychiatrist	50858 (57.42)	37712 (42.58)	21292 (24.04)	67278 (75.96)	46278 (52.25)	42292 (47.75)***
Others	42763 (25.17)	127139 (74.83)	27461 (16.16)	142441 (83.84)	25530 (15.03)	144372(84.97)

Abbreviations: MD- Medical Doctor, NTD- New Taiwan dollar.

***P<0.001.

**Table 6:** Multivariate logistic regression for having at least one potentially inappropriate medication in one clinic visit.

	Beers odds ratio	PIM-Taiwan odds ratio	PRISCUS odds ratio
Type of hospital visit			
Medical center	1	1	1
Others	0.92 (0.90–0.93)***	1.17 (1.15–1.20) ***	0.7 (0.69–0.72) ***
MD age			
< = 45 (years)	1	1	1
>45 (years)	1.06 (1.05–1.08) ***	1.15 (1.14–1.16) ***	0.94 (0.93–0.95) ***
MD gender			
Female	1	1	1
Male	1.12 (1.10–1.14)***	1.07 (1.04–1.09) ***	1.07 (1.05–1.10) ***
Specialty			
Others	1	1	1
Neuropsychiatrist	4.03 (3.96–4.10)***	1.73 (1.69–1.76)***	5.87 (5.76–5.98)***
Internist/family physician	1.68 (1.66–1.70)***	1.26 (1.24–1.28)***	1.72 (1.69–1.74) ***

***P<0.0001.

The top ten most frequently prescribed PIMs varied significantly in the three sets of criteria. The most frequent PIMs detected by the Beers criteria were psychotropic drugs, which accounted for 6 out of the top 10 medications. In contrast, the most frequent PIMs in the PIM-Taiwan criteria were first generation antihistamines (40%). In the PRISCUS criteria there was no dominant class of medication (Table 7).

**Table 7:** The leading ten potentially inappropriate medications (PIMs) identified in 2,428,222 prescribed medications.

Drug name and anatomical (ATC) code/prevalence of exposure (%)	Beers	PIM-Taiwan	PRISCUS
Piracetam (N06BX03)/1.46%			V
Metoclopramide (A03FA01)/1.18%	V		
Cimentidine (A02BA01)/1.01%		V	
Quietiapine (N05AH04)/0.91%	V		
Dipyridamole (B01AC07)/0.70%	V		
Lorazepam (N05BA06)/0.66%	V		
Zolpidem* (N05CF02)/0.64%	V		V
Digoxin (C01AA05)/0.60%			V
Clonazepam (N03AE01)/0.59%	V	V	
Estazolam (N05CD04)/0.44%	V		
Alprazolam (N05BA12)/0.44%	V		V
Doxazosin (C02CA04)/0.38%	V		V
Diclofenac (M01AB05)/0.36%	V		
Nifedipine (B01AC05)/0.34%			V
Pentoxifylline (C04AD03)/0.33%			V
Terazosin (G04CA03)/0.33%			V
Cyproheptadine (R06AX02)/0.29%		V	
Dexchlorpheniramine (R06AB02)/0.24%		V	
Ticlopidine (B01AC05)/0.24%		V	V
Baclofen (M03BX01)/0.23%		V	V
Mequitazine (R06AD07)/0.20%		V	
Chlorpheniramine (R06AB04)/0.18%		V	
Clorzoxazone (M03BB03)/0.17%		V	
Imipramine (N06AA02)/0.13%		V	

Beers criteria- 2012 version of Beers criteria, PIM-Taiwan criteria- potentially inappropriate medication Taiwan criteria.

*Zolpidem is considered as PIMs if daily dose is more than 5 mg in PRISCUS criteria but as PIMs regardless of daily dose in 2012 version of Beers criteria.

## Discussion

At least 60% of the Taiwanese HHSRs were prescribed with at least one PIM regardless of which set of criteria was used, however the prevalence of PIMs in the 2012 Beers criteria was highest (80%). HHSRs prescribed with PIMs were associated with polypharmacy, higher disease burden and higher healthcare utilization but age is the only discrepant factors between 3 sets of criteria. Internists/family physicians and neurologists/psychiatrists were more likely to prescribed PIMs than others. The leading PIMs detected were psychotropic drugs in the Beers criteria, and first generation antihistamine in the PIM-Taiwan criteria.

The annual personal prevalence rates of having at least one PIM in the three sets of criteria in this study were double those reported among HHSRs in the United States (31–38%) [17], [18] and eight countries in Europe (5.8–41.1%) [19]. The mean prevalence rate of PIMs among older Taiwanese with ambulatory care visits in 2001–2004 was reported to be 63.8% [20]. However, the 2003 version of Beers criteria was used in these studies. The prevalence of PIMs was reported to be 82.6% among Brazilian care home [21] residents based on the 2012 Beers criteria, and 24% in Germany based on the PRISCUS criteria [22]. There are several possible explanations for the relatively high prevalence of PIMs in our study population. First, the HHSRs were generally sicker than community patients with prescriptions for a higher number of medications. Since polypharmacy is a major determinant of PIM use, our findings were expected to be higher than most community studies. Second, we applied these criteria for all medications in the claims data without considering prescription duration. Third, limited data on the chronic conditions of our population were collected to exclude certain indications for appropriate use such as benzodiazepine for seizure control. Fourth, few clinically equivalent alternatives are available for certain PIMs such as benzodiazepine or Z-drug (e.g. zolpidem, zolpiclone, zaleplone) for insomnia. Finally, new medication classes have been added to the 2012 Beers criteria.

For the correlates the PIMs, we found that only age had discrepant results. Some studies have demonstrated that older age groups have less PIMs than younger age groups [18], [22]–[24], however other studies have not found this association [20], [25], [26]. In our analysis, inverse relationships were found between age and PIM use when the PIM-Taiwan and PRISCUS criteria were applied. In line with other studies [4], [18], [22], [27]–[29], we also demonstrated a relationship between higher disease burden, higher number of medications prescribed, and higher healthcare utilization with PIM use. Although these recipients had been regularly visited by home healthcare providers, these results suggested lack of continuity of care. Better continuity of care associated with lower prevalence of PIM use [30].

The differences in the medication lists and prescription preferences in Taiwan accounted for the variation in the prevalence of PIMs among three sets of criteria. In the 2012 Beers criteria, all benzodiazepines, both first and second generation antipsychotic agents, and all non-COX (cyclooxygenase) selective NSAIDs (non-steroidal anti-inflammatory drugs) are listed, however this is not the case in the PRISCUS and PIM-Taiwan criteria. The inclusion of entire medication classes as PIMs resulted in the highest prevalence of PIM use in the Beers criteria. In addition, the inclusion of entire medication classes may result in fewer available alternative medications to treat insomnia, dementia with behavioral and psychological symptoms and chronic pains.

The PIM-Taiwan and PRISCUS criteria had fewer statements than the 2012 Beers criteria and detected fewer PIMs. As we reported in our previous study, the availability of medications in the lists and number of statements are major determinants of PIM identification rate [7]. Several important newer medications such as Z-drugs are not included in the PIM-Taiwan criteria, and the medications accounting for the majority of PIMs in the other PIM lists should be included when the PIM-Taiwan list is updated.

This is the first study to address the problem of PIMs among older HHSRs in Taiwan. The results reveal several important issues. A significant portion (16–36%) of the HHSRs was prescribed with more than 10 kinds of PIM in one year. This indicates a real need for provider education to decrease the amount of PIM use. In Taiwan, HHS is usually provided by home healthcare nurses and family physicians [1]. The explicit PIM criteria can be used as a starting point to evaluate medication appropriateness [31], [32]. Although PIM criteria have been used for healthcare quality indicators in the United States and Europe, the method has not been adopted in Taiwan. Without administrative mandate for quality assurance, educational programs focus on these healthcare providers seems to be a reasonable choice to reduce the burden of polypharmacy and PIMs.

This study has several limitations. First, the claims data only listed three major diagnoses for each visit; therefore only statements for PIMs independent of chronic conditions were used. Second, because HHS in Taiwan were delivered to recipients in both community and nursing home, it is therefore difficult to compare our data with other studies based only on HHSRs lived in the community. Third, HHSRs requiring hospice care were not excluded from our study population. Some PIMs are considered to be appropriate for symptom relief for palliative or hospice care.

## Conclusion

The prevalence of PIMs was extremely high among Taiwanese HHSRs, especially when the 2012 Beers criteria were applied. Physicians treating patients with neuropsychiatric conditions prescribed PIMs most frequently. Physicians, home healthcare nurses and other home healthcare professionals should be familiar with these medication evaluation tools for medication reconciliation.
